# Comparison of propofol (1%) with admixture (1:1) of thiopentone (1.25%) and propofol (0.5%) for laryngeal mask airway insertion in children undergoing elective eye surgery: Double-masked randomized clinical trial

**DOI:** 10.4103/0019-5049.63641

**Published:** 2010

**Authors:** Renu Sinha, Dilip Shende, Rakesh Garg

**Affiliations:** Department of Anaesthesiology and Intensive Care, Rajendra Prasad Centre for Ophthalmic Sciences, All India Institute of Medical Sciences, Ansari Nagar, New Delhi - 110 029, India

**Keywords:** Admixture of propofol and thiopentone, LMA, paediatric

## Abstract

Intravenous propofol 1% has been the preferred agent for Laryngeal Mask Airway (LMA) insertion. Admixture of thiopentone 1.25% and propofol 0.5% (1:1) has been used by various authors for induction as well as insertion of LMA in adults. There is no previous report where this admixture has been used for insertion of LMA in children. This study has been designed to investigate whether this admixture can be a suitable alternative to propofol, in relation to ease of insertion of the LMA, haemodynamic stability, cost containment, pain on injection and recovery in children. In this randomized, double-masked study, 50 ASA grade 1 and 2 patients of age 3 – 15 years and weighing more than 10 kg were included. The patients were divided into two groups; the P group received propofol 1%, while the Ad group received an admixture of thiopentone 1.25% and propofol 0.5% (1:1). All the children were evaluated for incidence of apnoea, pain on injection, jaw relaxation, ease of LMA insertion, coughing, gagging, laryngospasm, involuntary limb movements, incidence of hypotension and recovery. The demographic data, incidence of apnoea, pain on injection, jaw relaxation, ease of LMA insertion, coughing, gagging and involuntary movements were comparable in both groups. In the P group recovery was faster as compared to the Ad group. The admixture was cost effective as compared to Propofol alone [Indian National Rupees (INR) 24.64 ± 7.62 vs. INR 48.75 ± 23.25] (*P* = 0.001)). Admixture of propofol and thiopentone was a cheap, safe and effective alternative to propofol alone, for LMA insertion in children.

## INTRODUCTION

The laryngeal mask airway (LMA) is commonly used for providing general anaesthesia in children, in anaesthetic practice, as it allows the maintenance of a clear airway while enabling the anaesthesiologist to keep both hands free and give full access of the operative field to the surgeon, especially in ophthalmic surgery.

Intravenous propofol (1%) has been the preferred induction agent for LMA insertion till date. It provides smooth induction with depression of airway reflexes, allowing easier insertion of LMA with a reduced incidence of side effects such as coughing, gagging or laryngospasm and rapid awakening.[1] However, propofol is expensive and causes pain at the injection site, which can be reduced by admixture with lignocaine or less conveniently by prior administration of thiopentone.[2,3]

Thiopentone 1.25% and propofol 0.5% admixture (1:1) has been used by various authors for induction,[4] as well as for insertion of LMA in adults,[1] without many side effects. However, there are no previous reports of the use of this admixture to facilitate LMA insertion in children. We planned this study to investigate whether the admixture of thiopentone and propofol (1:1) can be a suitable alternative to propofol in relation to ease the insertion of LMA and for haemodynamic stability, cost containment, pain on injection and recovery in paediatric patients.

## METHODS

Following approval of the institutional ethics committee, 50 children of ASA class I and II, belonging to either sex, of age 3 – 15 years, weighing more than 10 kg and scheduled for elective ophthalmic surgery, were included in the study. Children who were at risk of regurgitation, with known allergy to either agents, or with difficult airway were excluded. Children were prospectively, randomly allocated by the envelope method and the investigator and observer were blinded. In the preoperative room, with the child in the mother's lap, a 22 gauge cannula was inserted on the dorsum of the non-dominant hand.

Intravenous induction agents were prepared in 10 ml syringes, for Propofol group (P), 10 ml of propofol 1% was mixed with 10 mg of lignocaine (preservative-free) and for Admixture group (Ad), thiopentone 2.5% was mixed with propofol 1% in a 1:1 ratio to make it thiopentone 1.25% and propofol 0.5% per ml. They were indistinguishable from each other and strict measures were taken to avoid cross contamination. The admixture syringes were stored at operating theatre room temperature (21 to 23°C) and were used within 24 hours.

**Table 1:** Scoring system — Adverse response to airway manipulation[1] (Range: 1 – 3)

Parameters: Coughing, gagging, laryngospasm and involuntary limb movements		
1	Mild	Transient and minimal lasting < 5 seconds
2	Moderate	Lasted > 5 seconds, but resolved spontaneously within 20 seconds
3	Severe	Sustained > 20 seconds or required additional boluses of drugs

The parents were allowed in the operation suite and after application of standard monitoring, including heart rate (HR), electrocardiography (ECG), oxygen saturation (SpO_2_), and non-invasive blood pressure (NIBP); intravenous fentanyl 1.5 mcg/kg was administered 120 seconds prior to induction. The induction agent (0.25 ml/kg) was given over 30 seconds and the children were asked for pain or discomfort in the injection site till the children were conscious. An appropriate size LMA was inserted by an experienced anaesthesiologist (having > three years training in anaesthesia) blinded to drugs. Additional boluses of the induction agent were administered in 0.5 ml aliquots to deepen the anaesthesia, whenever required.

Adverse responses to airway manipulation, such as, coughing, gagging, laryngospasm and involuntary limb movement were graded as mild, moderate and severe [Table 1]. The ease of insertion of LMA and jaw relaxation were graded as excellent, satisfactory and poor [Table 2].[1]

Incidence of apnoea (absence of spontaneous respiration for > 20 seconds) was noted in the children and they were ventilated with 100%O_2_ before LMA insertion. After LMA insertion, anaesthesia was maintained with 33% oxygen in 67% nitrous oxide, and isoflurane, to maintain MAC 1.3. If the apnoea persisted or EtCO_2_ > 45 mmHg, ventilation was assisted manually. The occurrence of hypotension was noted and treated with ringer lactate at a rate of 4 ml/kg/hr. At the end of surgery, after LMA removal, recovery was evaluated by using the Aldrete score[5] (0 – 10 range) [Table 3]. If there were any incidences of postoperative nausea and vomiting and complications, they were noted. The acquisition cost of the drug was calculated using the mean dose required for LMA insertion in both groups.

The demographic data, dose of drug and Aldrete score were analysed using the analysis of variance (ANOVA) test. The incidence of adverse response to airway manipulation, such as, coughing, gagging, laryngospasm, involuntary limb movements, jaw relaxation and ease of LMA insertion was analysed using the Chi square test with Fisher exact test, wherever appropriate.

**Table 2:** Scoring system — Jaw relaxation and ease of laryngeal mask airway insertion[1] (Range: 1 – 3)

		
1	Excellent	No adverse responses
2	Satisfactory	Adverse response to airway manipulations, but not affecting the insertion of LMA
3	Poor	Moderate-to-severe adverse responses requiring additional boluses of drugs More than two attempts were required for LMA insertion

LMA: Laryngeal mask airway

## RESULTS

The demographic data were comparable in both the groups [Table 4]. Mean volume required for induction in the Ad group was 5.5 ± 1.7 ml and in the P group was 6.5 ± 3.1 ml [Tables 5], that is, 27.5 ± 8.5 mg of propofol in the Ad group and 65 ± 31 mg of propofol in the P group. Pain on injection was absent in both groups. The requirement of additional boluses of induction agent was also comparable and the mean rescue dose was comparable statistically. The incidence of adverse responses to airway manipulation, such as, coughing, gagging, laryngospasm, involuntary limb movements, incidence of apnoea and hypotension were comparable [Tables 5 and 6].

**Figure 1:**
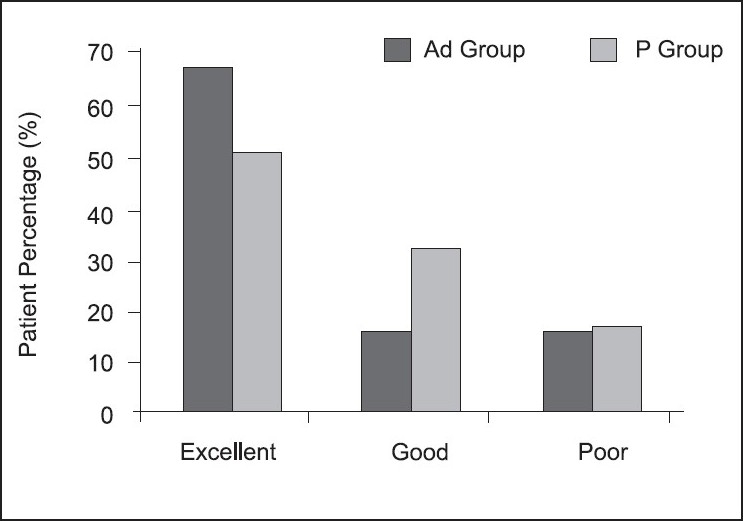
Jaw relaxation and ease of LMA insertion in the admixture group (Ad) and propofol group (P)

In the Ad group, excellent jaw relaxation and LMA insertion was seen in 17 (68%) patients and the same was observed in 13 (52%) patients in the P group [Figure 1]. Recovery was faster in the P group as compared to the Ad group (*P* < 0.001) [Table 5].

**Table 3:** Aldrete’s post-anaesthesia recovery scoring system[5] (Range: 0 – 10)

	Score
Activity	
Able to move all four limbs	2
Able to move only two limbs	1
Not able to move any limb	0
Respiration	
Able to breathe deeply and cough freely	2
Limited respiratory effort or dyspnoea	1
No spontaneous respiratory activity	0
Circulation	
SBP was ± 20% of the per anaesthetic level	2
SBP was between 20 and 50% of the pre-anaesthetic	1
level	0
SBP alteration was ± 50% or more	
Consciousness	
Fully alert, evidence by the ability to answer questions	2
Aroused only by calling their names	1
Auditing stimulation failed to elicit a response	0
Colour	
Obvious normal or pink colour	2
Pale, dusty or blotchy discolouration as well as jaundice	1
Frank cyanotic	0

**Table 4:** Demographic characteristics and duration of surgery (mean ± SD)

	Ad group (n = 25)	P group (n = 25)
Age (years)	8.24 ± 2.8	10.0 ± 3.8
Weight (kg)	21.92 ± 7.0	26.0 ± 12.3
Sex (M/F)	19/6	15/10
Duration of surgery (min)	34.56 ± 14.9	59.2 ± 28.9*

Ad – Admixture of thiopentone (1.25%) and propofol (0.5%) (1:1), P – Propofol (1%)

* *P* < 0.001

The total cost in the Ad group was Indian National Rupees (INR) 24.64 ± 7.62 and in the P group it was INR 48.75 ± 23.25 (*P* = 0.001) [Table 5].

## DISCUSSION

The laryngeal mask airway provides a direct connection to the tracheal airway without the need for laryngoscopy and tracheal intubation[6] and there is a decrease in the incidence of arterial oxygen desaturation, less airway stimulation and liberation of the anaesthesiologist to attend to other responsibilities. Changes in intraocular pressure are also blunted with the use of LMA as compared to endotracheal intubation.[7]

Successful insertion of LMA requires an adequate depth of anaesthesia by the use of either inhalation or intravenous agents to suppress pharyngeal and laryngeal reflexes.

To date, for LMA insertion, propofol is the agent of choice for intravenous induction, as it provides rapid induction with excellent jaw relaxation, but it has disadvantages such as pain at the injection site, involuntary limb movements, prolonged apnoea and hypotension.

**Table 5:** Dose, side effects of induction agent and recovery (mean ± SD) (%)

Assessment	Ad group (n = 25)	P group (n = 25)
Dose (ml)	5.5 ± 1.7	6.5 ± 3.1
No. of patients requiring additional boluses of induction agents	4 (16)	3 (12)
Incidence of apnoea	6 (24)	11 (44)
Incidence of hypotension	7 (28)	11 (44)
Time to reach aldrete 10 (min)	35.8 ± 12.2	10.72 ± 10.5*
PONV	0	0
Mean cost of induction agent per child (INR)	24.64 ± 7.62	48.75 ± 23.25*

Ad – Admixture of thiopentone (1.25%) and propofol (0.5%) (1:1), P – Propofol (1%), INR- Indian National Rupees,

* *P* ≤ 0.001, figures in parentheses are in percentages

**Table 6:** Incidence of adverse response to airway manipulation

Adverse effects	Ad group (n = 25)	P group (n = 25)	Statistical significance
Inadequate jaw relaxation	4 (16)	4 (16)	NS
Coughing	0	3 (12)	NS
Gagging	3 (12)	1 (4)	NS
Laryngospasm	1 (4)	0	NS
Involuntary limb movement	10 (40)	14 (56)	NS

Ad – Admixture of thiopentone (1.25%) and propofol (0.5%) (1:1), P – Propofol (1%),NS – not significant, figures in parentheses are in percentages

Thiopentone has advantage of painless injection and less incidence of hypotension, although it does not provide good jaw relaxation and can cause coughing, gagging and laryngospasm when used alone for LMA insertion.[8] It has been used with prior topical lignocaine spray to the posterior pharyngeal wall or co-induction with intravenous midazolam for LMA insertion in adults.[9]

Admixture of thiopentone and propofol is compatible and stable[10‐12] to its bactericidal properties, as it does not support the growth of micro-organisms despite the presence of nutrients in the admixture.[13] This admixture has a synergistic interaction[14] and does not prolong recovery when used for induction of anaesthesia and may reduce the incidence of convulsion. Cherin and Smiler took this admixture as an example of cost containment, while taking advantage of both the drugs, as it can be used for 24 hours if kept at operating room temperature (21 – 23°C), further decreasing wastage of drugs and thereby being more cost effective.[16] This admixture was used successfully for the induction of anaesthesia in adults.

Pain on injection can be considered a minor complication, but it may cause distress to the child and reduces acceptability of an otherwise useful agent. The cause of pain with propofol injection is due to the activation of kininogens[17] or to the free aqueous concentration of propofol in the emulsion.[18] Thiopentone reduces pain caused by propofol due to decrease in the release of kinins and change in the pH of the admixture. Jones D *et al*. showed that adding thiopentone to propofol could be as efficacious in preventing injection pain as mixing lignocaine 40 mg with 20-ml propofol. However, Lee TW *et al*. found thiopentone pre-treatment to be more effective than lignocaine.[2] In our study, none of the children in both the groups complained of pain on injection, which was similar to the study by Kau YC *et al*.[19] However, these studies were done in adults where pain evaluation is easier.

A study conducted by T. Goyagi *et al*. concluded that pre-treatment with fentanyl 2 mcg/kg^−1^ reduced the propofol requirement by 60% for LMA insertion,[20] hence, we used fentanyl 1.5 mcg/kg^−1^ before induction in both the groups and observed that the dose of propofol in paediatric patients was comparable to the adult dose used in the previous studies. A mean dose of propofol in the Ad group was half of that compared to the P group, suggesting an additive action of both the drugs, which was in confirmation with the studies of Yeo KSJ *et al*.[1] and Jones *et al*.[4] and explained by similar binding sites on the gamma-aminobutyric acid-A (GABA-A) receptors for propofol and barbiturates.[21] However, Naguib and Sari-Kouzel[14] demonstrated that sequential intravenous administration of thiopentone and propofol caused a synergistic interaction between them.

In our study, the condition for LMA insertion was excellent in 68% of the patients in the Ad group as compared to 52% in the P group, but this difference was statistically not significant while the incidence of various adverse responses to airway manipulation were similar in both the groups. Yeo KSJ *et al*.[1] found excellent conditions for LMA insertion in 65% of the patients in the P group as compared with 48.8% in the Ad group. This is in contrast to our finding and may be attributed to the paediatric population in our study.

There was a significant difference in the duration of surgery between the groups in our study, but this did not affect our results, as our main area of study was during induction and LMA insertion.

A fall in systolic blood pressure during propofol induction has been consistently reported in literature.[22] A decrease in the dose of propofol in the Ad group causes a decreased effect on afterload and the myocardium.[23] A decrease in the rate of administration of propofol decreases not only the dose required for induction, but also the degree of haemodynamic change.[24] In our study, the incidence of side effects such as the incidence of apnoea and hypotension were similar. Recovery in the P group was better as compared to the admixture group in our study, but this had no effect of the Post Anaesthesia Care Unit (PACU) discharge. Similar results were observed by Kern C *et al*.[25] in the adult population.

In our study, the admixture of thiopentone and propofol reduced the cost by half, as compared to propofol alone (INR 24.64 ± 7.62 vs. INR 48.75 ± 23.25), which can be of significance for paediatric population in developing countries, who come for repeated surgeries including examination under anaesthesia.

In conclusion, the admixture of thiopentone (1.25%) and propofol (0.5%) (1:1) is an acceptable and satisfactory alternative to propofol (1%) for induction of anaesthesia and LMA insertion in paediatric population.
